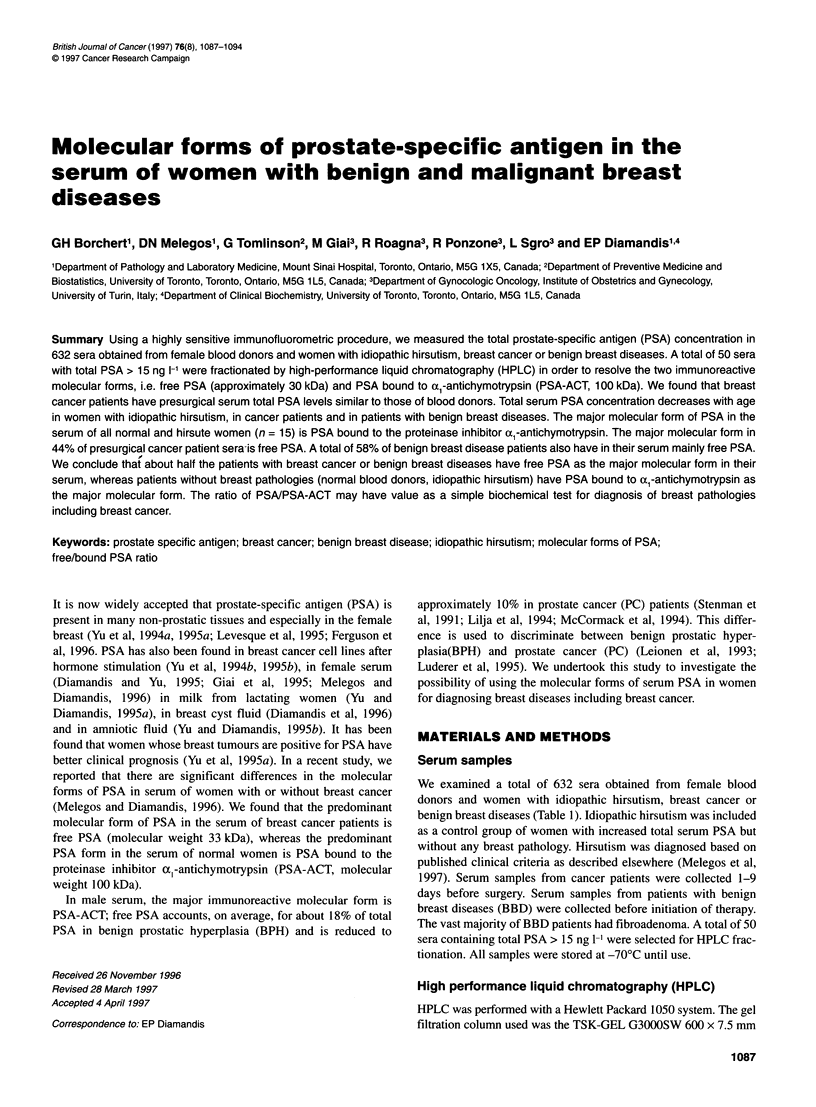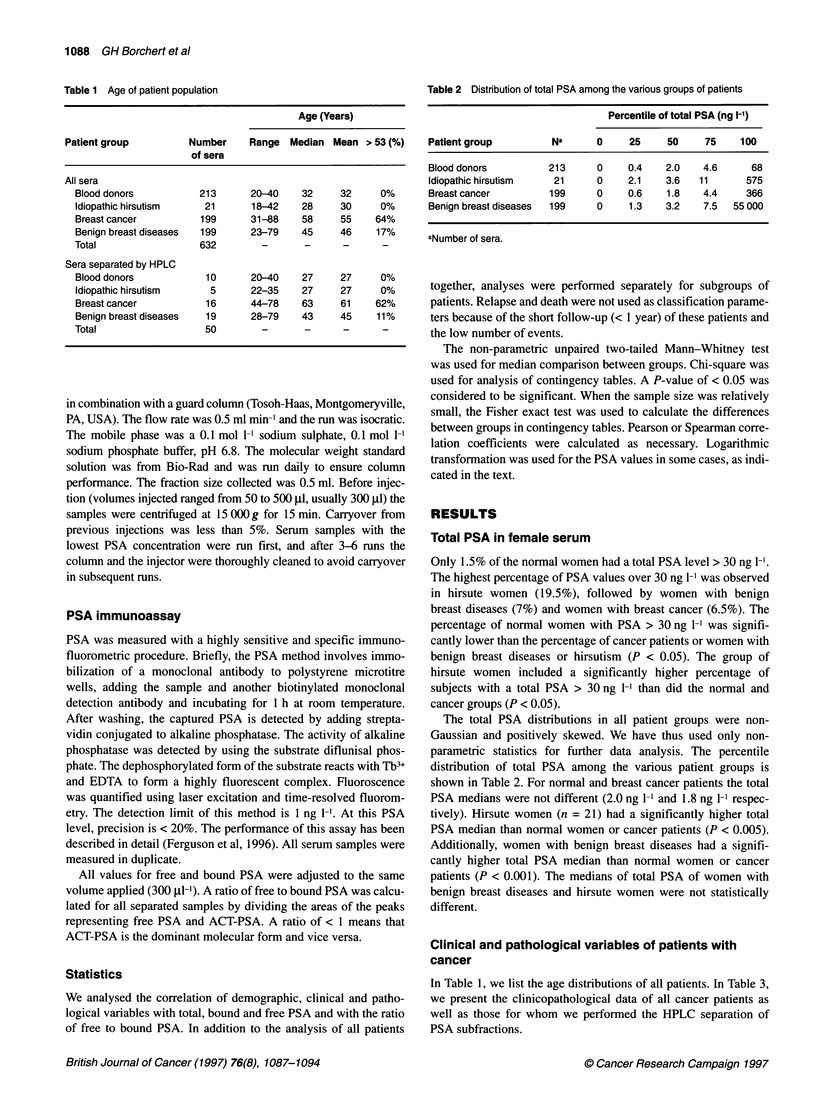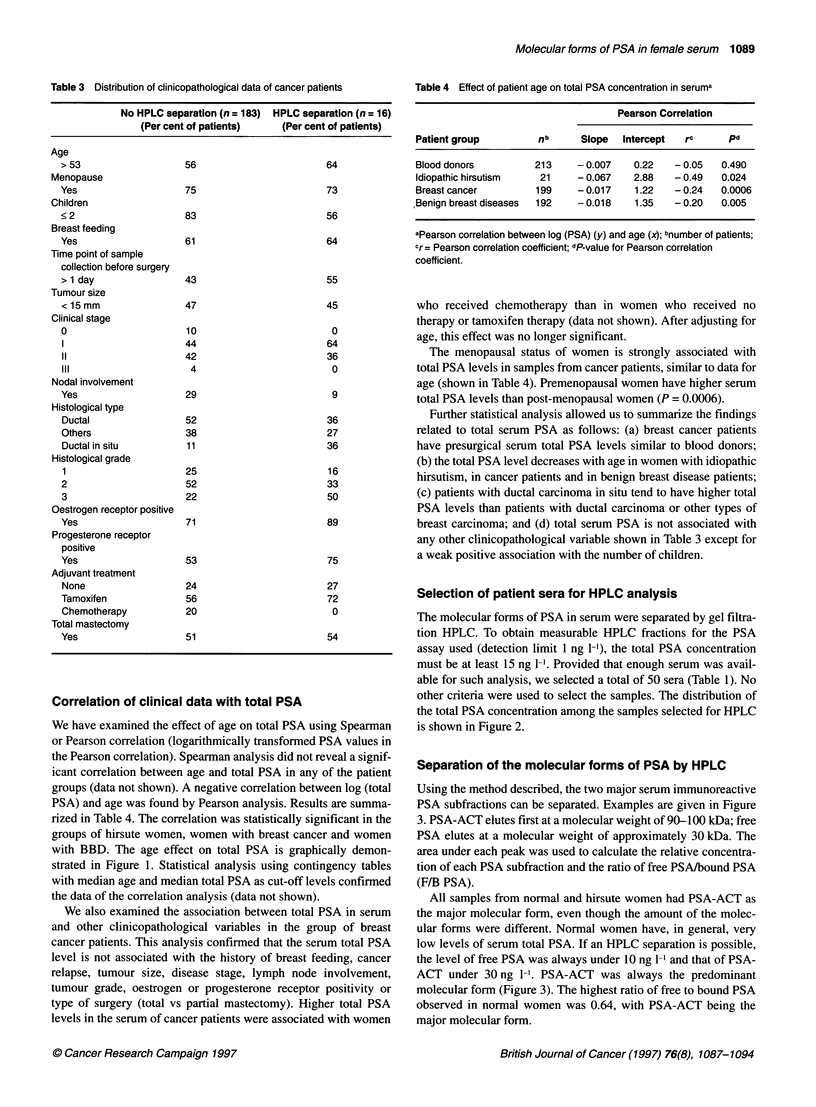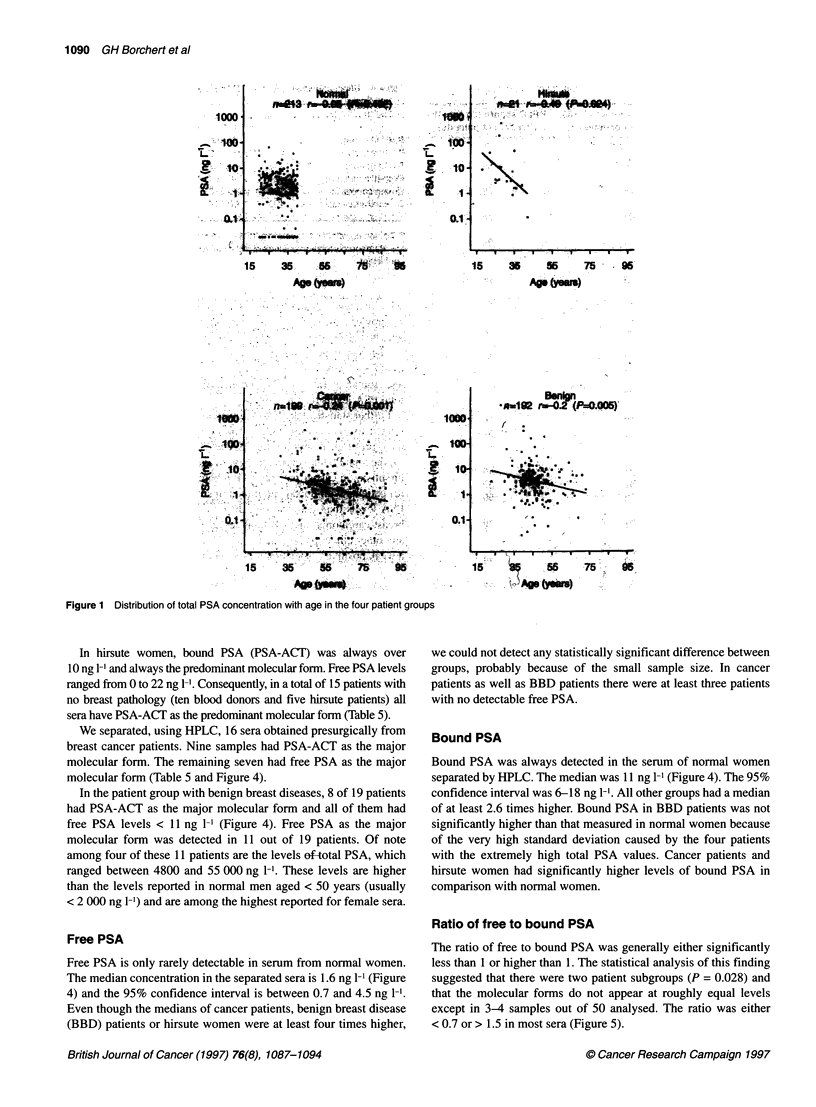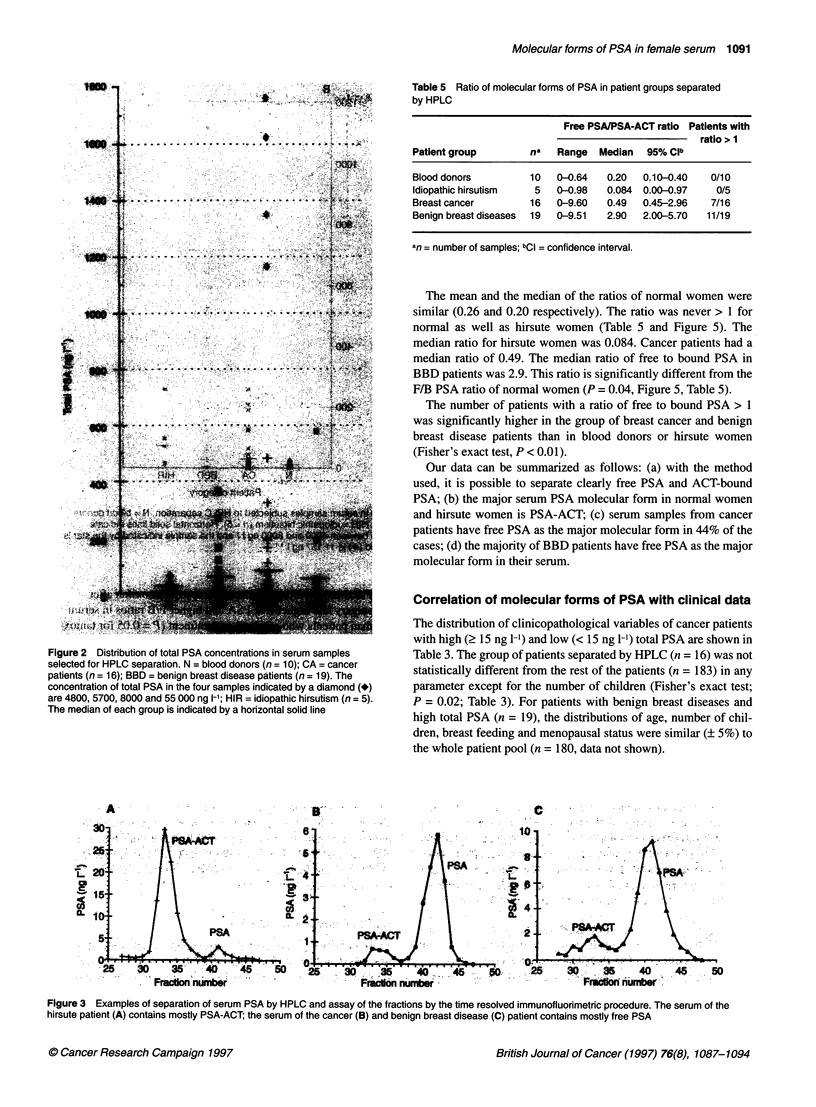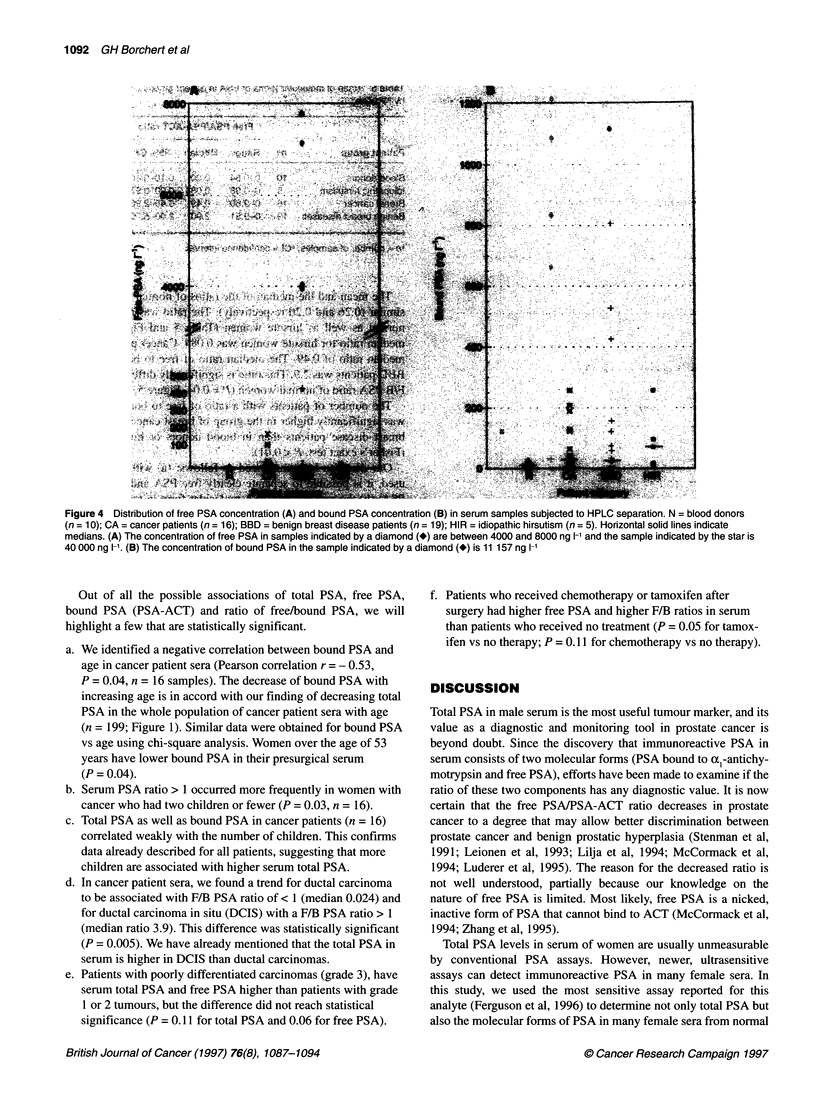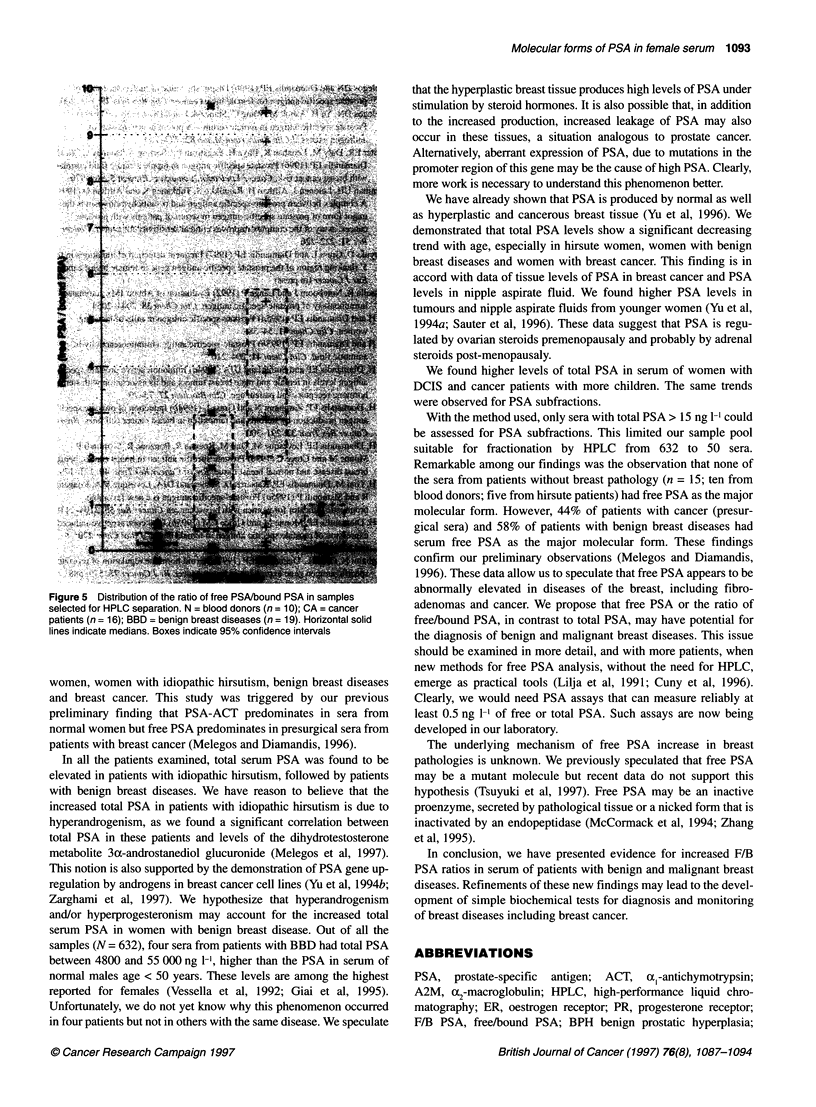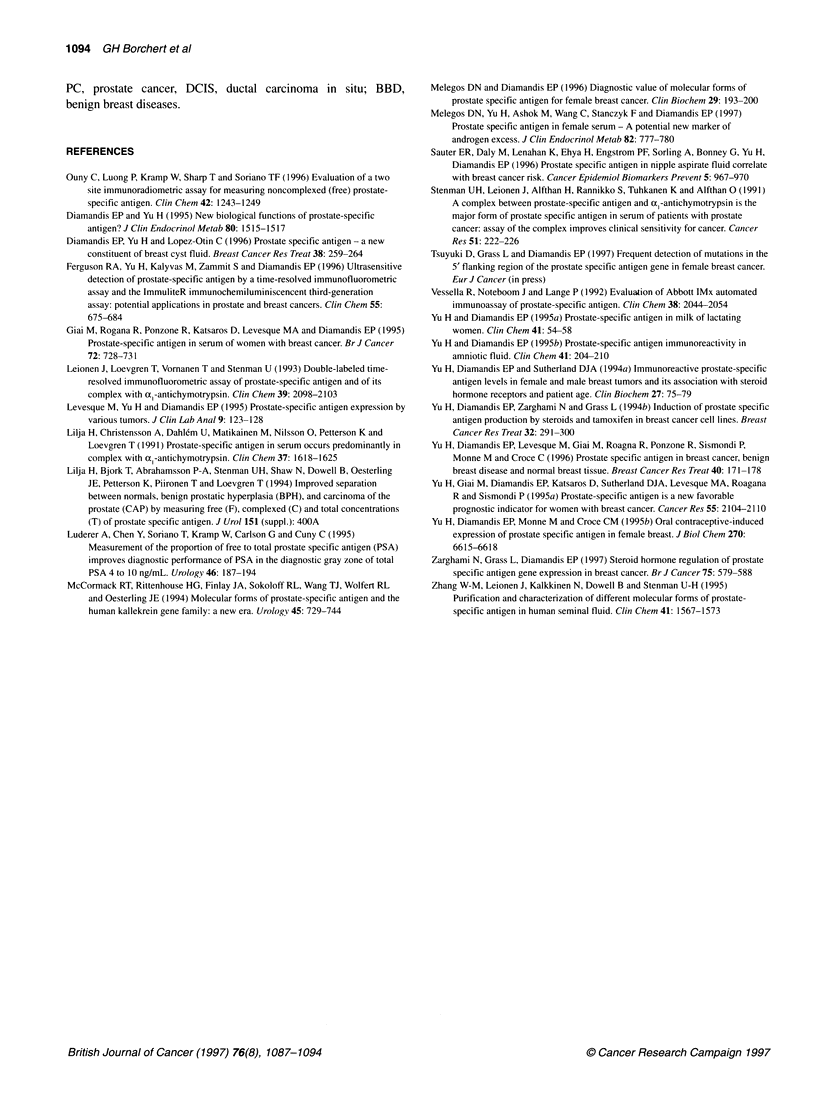# Molecular forms of prostate-specific antigen in the serum of women with benign and malignant breast diseases.

**DOI:** 10.1038/bjc.1997.512

**Published:** 1997

**Authors:** G. H. Borchert, D. N. Melegos, G. Tomlinson, M. Giai, R. Roagna, R. Ponzone, L. Sgro, E. P. Diamandis

**Affiliations:** Department of Pathology and Laboratory Medicine, Mount Sinai Hospital, Toronto, Ontario, Canada.

## Abstract

Using a highly sensitive immunofluorometric procedure, we measured the total prostate-specific antigen (PSA) concentration in 632 sera obtained from female blood donors and women with idiopathic hirsutism, breast cancer or benign breast diseases. A total of 50 sera with total PSA > 15 ng l(-1) were fractionated by high-performance liquid chromatography (HPLC) in order to resolve the two immunoreactive molecular forms, i.e. free PSA (approximately 30 kDa) and PSA bound to alpha1-antichymotrypsin (PSA-ACT, 100 kDa). We found that breast cancer patients have presurgical serum total PSA levels similar to those of blood donors. Total serum PSA concentration decreases with age in women with idiopathic hirsutism, in cancer patients and in patients with benign breast diseases. The major molecular form of PSA in the serum of all normal and hirsute women (n = 15) is PSA bound to the proteinase inhibitor alpha1-antichymotrypsin. The major molecular form in 44% of presurgical cancer patient sera is free PSA. A total of 58% of benign breast disease patients also have in their serum mainly free PSA. We conclude that about half the patients with breast cancer or benign breast diseases have free PSA as the major molecular form in their serum, whereas patients without breast pathologies (normal blood donors, idiopathic hirsutism) have PSA bound to alpha1-antichymotrypsin as the major molecular form. The ratio of PSA/PSA-ACT may have value as a simple biochemical test for diagnosis of breast pathologies including breast cancer.


British Journal of Cancer (1997) 76(8), 1087–1094
© 1997 Cancer Research Campaign

### Molecular forms of prostate-specific antigen in the serum of women with benign and malignant breast diseases

GH Borchert[1], DN Melegos[1], G Tomlinson[2], M Giai[3], R Roagna[3], R Ponzone[3], L Sgro[3] and EP Diamandis[1,4]

[1]Department of Pathology and Laboratory Medicine, Mount Sinai Hospital, Toronto, Ontario, M5G 1X5, Canada; [2]Department of Preventive Medicine and Biostatistics, University of Toronto, Toronto, Ontario, M5G 1L5, Canada; [3]Department of Gynocologic Oncology, Institute of Obstetrics and Gynecology, University of Turin, Italy; [4]Department of Clinical Biochemistry, University of Toronto, Toronto, Ontario, M5G 1L5, Canada

**Summary** Using a highly sensitive immunofluorometric procedure, we measured the total prostate-specific antigen (PSA) concentration in 632 sera obtained from female blood donors and women with idiopathic hirsutism, breast cancer or benign breast diseases. A total of 50 sera with total PSA > 15 ng $l^{-1}$ were fractionated by high-performance liquid chromatography (HPLC) in order to resolve the two immunoreactive molecular forms, i.e. free PSA (approximately 30 kDa) and PSA bound to $\alpha_1$-antichymotrypsin (PSA-ACT, 100 kDa). We found that breast cancer patients have presurgical serum total PSA levels similar to those of blood donors. Total serum PSA concentration decreases with age in women with idiopathic hirsutism, in cancer patients and in patients with benign breast diseases. The major molecular form of PSA in the serum of all normal and hirsute women ($n$ = 15) is PSA bound to the proteinase inhibitor $\alpha_1$-antichymotrypsin. The major molecular form in 44% of presurgical cancer patient sera is free PSA. A total of 58% of benign breast disease patients also have in their serum mainly free PSA. We conclude that about half the patients with breast cancer or benign breast diseases have free PSA as the major molecular form in their serum, whereas patients without breast pathologies (normal blood donors, idiopathic hirsutism) have PSA bound to $\alpha_1$-antichymotrypsin as the major molecular form. The ratio of PSA/PSA-ACT may have value as a simple biochemical test for diagnosis of breast pathologies including breast cancer.

**Keywords:** prostate specific antigen; breast cancer; benign breast disease; idiopathic hirsutism; molecular forms of PSA; free/bound PSA ratio

It is now widely accepted that prostate-specific antigen (PSA) is present in many non-prostatic tissues and especially in the female breast (Yu et al, 1994*a*, 1995*a*; Levesque et al, 1995; Ferguson et al, 1996. PSA has also been found in breast cancer cell lines after hormone stimulation (Yu et al, 1994*b*, 1995*b*), in female serum (Diamandis and Yu, 1995; Giai et al, 1995; Melegos and Diamandis, 1996) in milk from lactating women (Yu and Diamandis, 1995*a*), in breast cyst fluid (Diamandis et al, 1996) and in amniotic fluid (Yu and Diamandis, 1995*b*). It has been found that women whose breast tumours are positive for PSA have better clinical prognosis (Yu et al, 1995*a*). In a recent study, we reported that there are significant differences in the molecular forms of PSA in serum of women with or without breast cancer (Melegos and Diamandis, 1996). We found that the predominant molecular form of PSA in the serum of breast cancer patients is free PSA (molecular weight 33 kDa), whereas the predominant PSA form in the serum of normal women is PSA bound to the proteinase inhibitor $\alpha_1$-antichymotrypsin (PSA-ACT, molecular weight 100 kDa).

In male serum, the major immunoreactive molecular form is PSA-ACT; free PSA accounts, on average, for about 18% of total PSA in benign prostatic hyperplasia (BPH) and is reduced to approximately 10% in prostate cancer (PC) patients (Stenman et al, 1991; Lilja et al, 1994; McCormack et al, 1994). This difference is used to discriminate between benign prostatic hyperplasia(BPH) and prostate cancer (PC) (Leionen et al, 1993; Luderer et al, 1995). We undertook this study to investigate the possibility of using the molecular forms of serum PSA in women for diagnosing breast diseases including breast cancer.

*Received 26 November 1996*
*Revised 28 March 1997*
*Accepted 4 April 1997*

*Correspondence to:* EP Diamandis

### MATERIALS AND METHODS

### Serum samples

We examined a total of 632 sera obtained from female blood donors and women with idiopathic hirsutism, breast cancer or benign breast diseases (Table 1). Idiopathic hirsutism was included as a control group of women with increased total serum PSA but without any breast pathology. Hirsutism was diagnosed based on published clinical criteria as described elsewhere (Melegos et al, 1997). Serum samples from cancer patients were collected 1–9 days before surgery. Serum samples from patients with benign breast diseases (BBD) were collected before initiation of therapy. The vast majority of BBD patients had fibroadenoma. A total of 50 sera containing total PSA > 15 ng $l^{-1}$ were selected for HPLC fractionation. All samples were stored at –70°C until use.

### High performance liquid chromatography (HPLC)

HPLC was performed with a Hewlett Packard 1050 system. The gel filtration column used was the TSK-GEL G3000SW 600 × 7.5 mm

**Table 1** Age of patient population

| Patient group | Number of sera | Age (Years) Range | Median | Mean | > 53 (%) |
|---|---|---|---|---|---|
| All sera | | | | | |
| Blood donors | 213 | 20–40 | 32 | 32 | 0% |
| Idiopathic hirsutism | 21 | 18–42 | 28 | 30 | 0% |
| Breast cancer | 199 | 31–88 | 58 | 55 | 64% |
| Benign breast diseases | 199 | 23–79 | 45 | 46 | 17% |
| Total | 632 | – | – | – | – |
| Sera separated by HPLC | | | | | |
| Blood donors | 10 | 20–40 | 27 | 27 | 0% |
| Idiopathic hirsutism | 5 | 22–35 | 27 | 27 | 0% |
| Breast cancer | 16 | 44–78 | 63 | 61 | 62% |
| Benign breast diseases | 19 | 28–79 | 43 | 45 | 11% |
| Total | 50 | – | – | – | – |

**Table 2** Distribution of total PSA among the various groups of patients

| Patient group | N[a] | Percentile of total PSA (ng $l^{-1}$) 0 | 25 | 50 | 75 | 100 |
|---|---|---|---|---|---|---|
| Blood donors | 213 | 0 | 0.4 | 2.0 | 4.6 | 68 |
| Idiopathic hirsutism | 21 | 0 | 2.1 | 3.6 | 11 | 575 |
| Breast cancer | 199 | 0 | 0.6 | 1.8 | 4.4 | 366 |
| Benign breast diseases | 199 | 0 | 1.3 | 3.2 | 7.5 | 55 000 |

[a]Number of sera.

in combination with a guard column (Tosoh-Haas, Montgomeryville, PA, USA). The flow rate was 0.5 ml $min^{-1}$ and the run was isocratic. The mobile phase was a 0.1 mol $l^{-1}$ sodium sulphate, 0.1 mol $l^{-1}$ sodium phosphate buffer, pH 6.8. The molecular weight standard solution was from Bio-Rad and was run daily to ensure column performance. The fraction size collected was 0.5 ml. Before injection (volumes injected ranged from 50 to 500 μl, usually 300 μl) the samples were centrifuged at 15 000 *g* for 15 min. Carryover from previous injections was less than 5%. Serum samples with the lowest PSA concentration were run first, and after 3–6 runs the column and the injector were thoroughly cleaned to avoid carryover in subsequent runs.

### PSA immunoassay

PSA was measured with a highly sensitive and specific immunofluorometric procedure. Briefly, the PSA method involves immobilization of a monoclonal antibody to polystyrene microtitre wells, adding the sample and another biotinylated monoclonal detection antibody and incubating for 1 h at room temperature. After washing, the captured PSA is detected by adding streptavidin conjugated to alkaline phosphatase. The activity of alkaline phosphatase was detected by using the substrate diflunisal phosphate. The dephosphorylated form of the substrate reacts with $Tb^{3+}$ and EDTA to form a highly fluorescent complex. Fluoroscence was quantified using laser excitation and time-resolved fluorometry. The detection limit of this method is 1 ng $l^{-1}$. At this PSA level, precision is < 20%. The performance of this assay has been described in detail (Ferguson et al, 1996). All serum samples were measured in duplicate.

All values for free and bound PSA were adjusted to the same volume applied (300 μ$l^{-1}$). A ratio of free to bound PSA was calculated for all separated samples by dividing the areas of the peaks representing free PSA and ACT-PSA. A ratio of < 1 means that ACT-PSA is the dominant molecular form and vice versa.

### Statistics

We analysed the correlation of demographic, clinical and pathological variables with total, bound and free PSA and with the ratio of free to bound PSA. In addition to the analysis of all patients together, analyses were performed separately for subgroups of patients. Relapse and death were not used as classification parameters because of the short follow-up (< 1 year) of these patients and the low number of events.

The non-parametric unpaired two-tailed Mann–Whitney test was used for median comparison between groups. Chi-square was used for analysis of contingency tables. A *P*-value of < 0.05 was considered to be significant. When the sample size was relatively small, the Fisher exact test was used to calculate the differences between groups in contingency tables. Pearson or Spearman correlation coefficients were calculated as necessary. Logarithmic transformation was used for the PSA values in some cases, as indicated in the text.

### RESULTS

### Total PSA in female serum

Only 1.5% of the normal women had a total PSA level > 30 ng $l^{-1}$. The highest percentage of PSA values over 30 ng $l^{-1}$ was observed in hirsute women (19.5%), followed by women with benign breast diseases (7%) and women with breast cancer (6.5%). The percentage of normal women with PSA > 30 ng $l^{-1}$ was significantly lower than the percentage of cancer patients or women with benign breast diseases or hirsutism ($P < 0.05$). The group of hirsute women included a significantly higher percentage of subjects with a total PSA > 30 ng $l^{-1}$ than did the normal and cancer groups ($P < 0.05$).

The total PSA distributions in all patient groups were non-Gaussian and positively skewed. We have thus used only non-parametric statistics for further data analysis. The percentile distribution of total PSA among the various patient groups is shown in Table 2. For normal and breast cancer patients the total PSA medians were not different (2.0 ng $l^{-1}$ and 1.8 ng $l^{-1}$ respectively). Hirsute women ($n = 21$) had a significantly higher total PSA median than normal women or cancer patients ($P < 0.005$). Additionally, women with benign breast diseases had a significantly higher total PSA median than normal women or cancer patients ($P < 0.001$). The medians of total PSA of women with benign breast diseases and hirsute women were not statistically different.

### Clinical and pathological variables of patients with cancer

In Table 1, we list the age distributions of all patients. In Table 3, we present the clinicopathological data of all cancer patients as well as those for whom we performed the HPLC separation of PSA subfractions.

© Cancer Research Campaign 1997

**Table 3** Distribution of clinicopathological data of cancer patients

| | No HPLC separation ($n$ = 183) (Per cent of patients) | HPLC separation ($n$ = 16) (Per cent of patients) |
|---|---|---|
| Age | | |
| > 53 | 56 | 64 |
| Menopause | | |
| Yes | 75 | 73 |
| Children | | |
| ≤ 2 | 83 | 56 |
| Breast feeding | | |
| Yes | 61 | 64 |
| Time point of sample collection before surgery | | |
| > 1 day | 43 | 55 |
| Tumour size | | |
| < 15 mm | 47 | 45 |
| Clinical stage | | |
| 0 | 10 | 0 |
| I | 44 | 64 |
| II | 42 | 36 |
| III | 4 | 0 |
| Nodal involvement | | |
| Yes | 29 | 9 |
| Histological type | | |
| Ductal | 52 | 36 |
| Others | 38 | 27 |
| Ductal in situ | 11 | 36 |
| Histological grade | | |
| 1 | 25 | 16 |
| 2 | 52 | 33 |
| 3 | 22 | 50 |
| Oestrogen receptor positive | | |
| Yes | 71 | 89 |
| Progesterone receptor positive | | |
| Yes | 53 | 75 |
| Adjuvant treatment | | |
| None | 24 | 27 |
| Tamoxifen | 56 | 72 |
| Chemotherapy | 20 | 0 |
| Total mastectomy | | |
| Yes | 51 | 54 |

### Correlation of clinical data with total PSA

We have examined the effect of age on total PSA using Spearman or Pearson correlation (logarithmically transformed PSA values in the Pearson correlation). Spearman analysis did not reveal a significant correlation between age and total PSA in any of the patient groups (data not shown). A negative correlation between log (total PSA) and age was found by Pearson analysis. Results are summarized in Table 4. The correlation was statistically significant in the groups of hirsute women, women with breast cancer and women with BBD. The age effect on total PSA is graphically demonstrated in Figure 1. Statistical analysis using contingency tables with median age and median total PSA as cut-off levels confirmed the data of the correlation analysis (data not shown).

We also examined the association between total PSA in serum and other clinicopathological variables in the group of breast cancer patients. This analysis confirmed that the serum total PSA level is not associated with the history of breast feeding, cancer relapse, tumour size, disease stage, lymph node involvement, tumour grade, oestrogen or progesterone receptor positivity or type of surgery (total vs partial mastectomy). Higher total PSA levels in the serum of cancer patients were associated with women who received chemotherapy than in women who received no therapy or tamoxifen therapy (data not shown). After adjusting for age, this effect was no longer significant.

**Table 4** Effect of patient age on total PSA concentration in serum[a]

| | | Pearson Correlation | | | |
|---|---|---|---|---|---|
| Patient group | $n^b$ | Slope | Intercept | $r^c$ | $P^d$ |
| Blood donors | 213 | − 0.007 | 0.22 | − 0.05 | 0.490 |
| Idiopathic hirsutism | 21 | − 0.067 | 2.88 | − 0.49 | 0.024 |
| Breast cancer | 199 | − 0.017 | 1.22 | − 0.24 | 0.0006 |
| Benign breast diseases | 192 | − 0.018 | 1.35 | − 0.20 | 0.005 |

[a]Pearson correlation between log (PSA) ($y$) and age ($x$); [b]number of patients; [c]$r$ = Pearson correlation coefficient; [d]$P$-value for Pearson correlation coefficient.

The menopausal status of women is strongly associated with total PSA levels in samples from cancer patients, similar to data for age (shown in Table 4). Premenopausal women have higher serum total PSA levels than post-menopausal women ($P$ = 0.0006).

Further statistical analysis allowed us to summarize the findings related to total serum PSA as follows: (a) breast cancer patients have presurgical serum total PSA levels similar to blood donors; (b) the total PSA level decreases with age in women with idiopathic hirsutism, in cancer patients and in benign breast disease patients; (c) patients with ductal carcinoma in situ tend to have higher total PSA levels than patients with ductal carcinoma or other types of breast carcinoma; and (d) total serum PSA is not associated with any other clinicopathological variable shown in Table 3 except for a weak positive association with the number of children.

### Selection of patient sera for HPLC analysis

The molecular forms of PSA in serum were separated by gel filtration HPLC. To obtain measurable HPLC fractions for the PSA assay used (detection limit 1 ng $l^{-1}$), the total PSA concentration must be at least 15 ng $l^{-1}$. Provided that enough serum was available for such analysis, we selected a total of 50 sera (Table 1). No other criteria were used to select the samples. The distribution of the total PSA concentration among the samples selected for HPLC is shown in Figure 2.

### Separation of the molecular forms of PSA by HPLC

Using the method described, the two major serum immunoreactive PSA subfractions can be separated. Examples are given in Figure 3. PSA-ACT elutes first at a molecular weight of 90–100 kDa; free PSA elutes at a molecular weight of approximately 30 kDa. The area under each peak was used to calculate the relative concentration of each PSA subfraction and the ratio of free PSA/bound PSA (F/B PSA).

All samples from normal and hirsute women had PSA-ACT as the major molecular form, even though the amount of the molecular forms were different. Normal women have, in general, very low levels of serum total PSA. If an HPLC separation is possible, the level of free PSA was always under 10 ng $l^{-1}$ and that of PSA-ACT under 30 ng $l^{-1}$. PSA-ACT was always the predominant molecular form (Figure 3). The highest ratio of free to bound PSA observed in normal women was 0.64, with PSA-ACT being the major molecular form.

© Cancer Research Campaign 1997

**Figure 1** Distribution of total PSA concentration with age in the four patient groups

In hirsute women, bound PSA (PSA-ACT) was always over 10 ng $l^{-1}$ and always the predominant molecular form. Free PSA levels ranged from 0 to 22 ng $l^{-1}$. Consequently, in a total of 15 patients with no breast pathology (ten blood donors and five hirsute patients) all sera have PSA-ACT as the predominant molecular form (Table 5).

We separated, using HPLC, 16 sera obtained presurgically from breast cancer patients. Nine samples had PSA-ACT as the major molecular form. The remaining seven had free PSA as the major molecular form (Table 5 and Figure 4).

In the patient group with benign breast diseases, 8 of 19 patients had PSA-ACT as the major molecular form and all of them had free PSA levels < 11 ng $l^{-1}$ (Figure 4). Free PSA as the major molecular form was detected in 11 out of 19 patients. Of note among four of these 11 patients are the levels of total PSA, which ranged between 4800 and 55 000 ng $l^{-1}$. These levels are higher than the levels reported in normal men aged < 50 years (usually < 2 000 ng $l^{-1}$) and are among the highest reported for female sera.

### Free PSA

Free PSA is only rarely detectable in serum from normal women. The median concentration in the separated sera is 1.6 ng $l^{-1}$ (Figure 4) and the 95% confidence interval is between 0.7 and 4.5 ng $l^{-1}$. Even though the medians of cancer patients, benign breast disease (BBD) patients or hirsute women were at least four times higher, we could not detect any statistically significant difference between groups, probably because of the small sample size. In cancer patients as well as BBD patients there were at least three patients with no detectable free PSA.

### Bound PSA

Bound PSA was always detected in the serum of normal women separated by HPLC. The median was 11 ng $l^{-1}$ (Figure 4). The 95% confidence interval was 6–18 ng $l^{-1}$. All other groups had a median of at least 2.6 times higher. Bound PSA in BBD patients was not significantly higher than that measured in normal women because of the very high standard deviation caused by the four patients with the extremely high total PSA values. Cancer patients and hirsute women had significantly higher levels of bound PSA in comparison with normal women.

### Ratio of free to bound PSA

The ratio of free to bound PSA was generally either significantly less than 1 or higher than 1. The statistical analysis of this finding suggested that there were two patient subgroups ($P = 0.028$) and that the molecular forms do not appear at roughly equal levels except in 3–4 samples out of 50 analysed. The ratio was either < 0.7 or > 1.5 in most sera (Figure 5).

© Cancer Research Campaign 1997

**Figure 2** Distribution of total PSA concentrations in serum samples selected for HPLC separation. N = blood donors ($n$ = 10); CA = cancer patients ($n$ = 16); BBD = benign breast disease patients ($n$ = 19). The concentration of total PSA in the four samples indicated by a diamond (◆) are 4800, 5700, 8000 and 55 000 ng $l^{-1}$; HIR = idiopathic hirsutism ($n$ = 5). The median of each group is indicated by a horizontal solid line

**Table 5** Ratio of molecular forms of PSA in patient groups separated by HPLC

| Patient group | $n$[a] | Free PSA/PSA-ACT ratio Range | Median | 95% CI[b] | Patients with ratio > 1 |
|---|---|---|---|---|---|
| Blood donors | 10 | 0–0.64 | 0.20 | 0.10–0.40 | 0/10 |
| Idiopathic hirsutism | 5 | 0–0.98 | 0.084 | 0.00–0.97 | 0/5 |
| Breast cancer | 16 | 0–9.60 | 0.49 | 0.45–2.96 | 7/16 |
| Benign breast diseases | 19 | 0–9.51 | 2.90 | 2.00–5.70 | 11/19 |

[a]$n$ = number of samples; [b]CI = confidence interval.

The mean and the median of the ratios of normal women were similar (0.26 and 0.20 respectively). The ratio was never > 1 for normal as well as hirsute women (Table 5 and Figure 5). The median ratio for hirsute women was 0.084. Cancer patients had a median ratio of 0.49. The median ratio of free to bound PSA in BBD patients was 2.9. This ratio is significantly different from the F/B PSA ratio of normal women ($P$ = 0.04, Figure 5, Table 5).

The number of patients with a ratio of free to bound PSA > 1 was significantly higher in the group of breast cancer and benign breast disease patients than in blood donors or hirsute women (Fisher's exact test, $P < 0.01$).

Our data can be summarized as follows: (a) with the method used, it is possible to separate clearly free PSA and ACT-bound PSA; (b) the major serum PSA molecular form in normal women and hirsute women is PSA-ACT; (c) serum samples from cancer patients have free PSA as the major molecular form in 44% of the cases; (d) the majority of BBD patients have free PSA as the major molecular form in their serum.

### Correlation of molecular forms of PSA with clinical data

The distribution of clinicopathological variables of cancer patients with high (≥ 15 ng $l^{-1}$) and low (< 15 ng $l^{-1}$) total PSA are shown in Table 3. The group of patients separated by HPLC ($n$ = 16) was not statistically different from the rest of the patients ($n$ = 183) in any parameter except for the number of children (Fisher's exact test; $P$ = 0.02; Table 3). For patients with benign breast diseases and high total PSA ($n$ = 19), the distributions of age, number of children, breast feeding and menopausal status were similar (± 5%) to the whole patient pool ($n$ = 180, data not shown).

**Figure 3** Examples of separation of serum PSA by HPLC and assay of the fractions by the time resolved immunofluorimetric procedure. The serum of the hirsute patient (**A**) contains mostly PSA-ACT; the serum of the cancer (**B**) and benign breast disease (**C**) patient contains mostly free PSA

**Figure 4** Distribution of free PSA concentration (**A**) and bound PSA concentration (**B**) in serum samples subjected to HPLC separation. N = blood donors ($n$ = 10); CA = cancer patients ($n$ = 16); BBD = benign breast disease patients ($n$ = 19); HIR = idiopathic hirsutism ($n$ = 5). Horizontal solid lines indicate medians. (**A**) The concentration of free PSA in samples indicated by a diamond (◆) are between 4000 and 8000 ng $l^{-1}$ and the sample indicated by the star is 40 000 ng $l^{-1}$. (**B**) The concentration of bound PSA in the sample indicated by a diamond (◆) is 11 157 ng $l^{-1}$

Out of all the possible associations of total PSA, free PSA, bound PSA (PSA-ACT) and ratio of free/bound PSA, we will highlight a few that are statistically significant.

a. We identified a negative correlation between bound PSA and age in cancer patient sera (Pearson correlation $r = -0.53$, $P = 0.04$, $n = 16$ samples). The decrease of bound PSA with increasing age is in accord with our finding of decreasing total PSA in the whole population of cancer patient sera with age ($n = 199$; Figure 1). Similar data were obtained for bound PSA vs age using chi-square analysis. Women over the age of 53 years have lower bound PSA in their presurgical serum ($P = 0.04$).
b. Serum PSA ratio > 1 occurred more frequently in women with cancer who had two children or fewer ($P = 0.03$, $n = 16$).
c. Total PSA as well as bound PSA in cancer patients ($n = 16$) correlated weakly with the number of children. This confirms data already described for all patients, suggesting that more children are associated with higher serum total PSA.
d. In cancer patient sera, we found a trend for ductal carcinoma to be associated with F/B PSA ratio of < 1 (median 0.024) and for ductal carcinoma in situ (DCIS) with a F/B PSA ratio > 1 (median ratio 3.9). This difference was statistically significant ($P = 0.005$). We have already mentioned that the total PSA in serum is higher in DCIS than ductal carcinomas.
e. Patients with poorly differentiated carcinomas (grade 3), have serum total PSA and free PSA higher than patients with grade 1 or 2 tumours, but the difference did not reach statistical significance ($P = 0.11$ for total PSA and 0.06 for free PSA).
f. Patients who received chemotherapy or tamoxifen after surgery had higher free PSA and higher F/B ratios in serum than patients who received no treatment ($P = 0.05$ for tamoxifen vs no therapy; $P = 0.11$ for chemotherapy vs no therapy).

### DISCUSSION

Total PSA in male serum is the most useful tumour marker, and its value as a diagnostic and monitoring tool in prostate cancer is beyond doubt. Since the discovery that immunoreactive PSA in serum consists of two molecular forms (PSA bound to $\alpha_1$-antichymotrypsin and free PSA), efforts have been made to examine if the ratio of these two components has any diagnostic value. It is now certain that the free PSA/PSA-ACT ratio decreases in prostate cancer to a degree that may allow better discrimination between prostate cancer and benign prostatic hyperplasia (Stenman et al, 1991; Leionen et al, 1993; Lilja et al, 1994; McCormack et al, 1994; Luderer et al, 1995). The reason for the decreased ratio is not well understood, partially because our knowledge on the nature of free PSA is limited. Most likely, free PSA is a nicked, inactive form of PSA that cannot bind to ACT (McCormack et al, 1994; Zhang et al, 1995).

Total PSA levels in serum of women are usually unmeasurable by conventional PSA assays. However, newer, ultrasensitive assays can detect immunoreactive PSA in many female sera. In this study, we used the most sensitive assay reported for this analyte (Ferguson et al, 1996) to determine not only total PSA but also the molecular forms of PSA in many female sera from normal

© Cancer Research Campaign 1997

**Figure 5** Distribution of the ratio of free PSA/bound PSA in samples selected for HPLC separation. N = blood donors (*n* = 10); CA = cancer patients (*n* = 16); BBD = benign breast diseases (*n* = 19). Horizontal solid lines indicate medians. Boxes indicate 95% confidence intervals women, women with idiopathic hirsutism, benign breast diseases and breast cancer. This study was triggered by our previous preliminary finding that PSA-ACT predominates in sera from normal women but free PSA predominates in presurgical sera from patients with breast cancer (Melegos and Diamandis, 1996).

In all the patients examined, total serum PSA was found to be elevated in patients with idiopathic hirsutism, followed by patients with benign breast diseases. We have reason to believe that the increased total PSA in patients with idiopathic hirsutism is due to hyperandrogenism, as we found a significant correlation between total PSA in these patients and levels of the dihydrotestosterone metabolite 3α-androstanediol glucuronide (Melegos et al, 1997). This notion is also supported by the demonstration of PSA gene up-regulation by androgens in breast cancer cell lines (Yu et al, 1994*b*; Zarghami et al, 1997). We hypothesize that hyperandrogenism and/or hyperprogesteronism may account for the increased total serum PSA in women with benign breast disease. Out of all the samples (*N* = 632), four sera from patients with BBD had total PSA between 4800 and 55 000 ng $l^{-1}$, higher than the PSA in serum of normal males age < 50 years. These levels are among the highest reported for females (Vessella et al, 1992; Giai et al, 1995). Unfortunately, we do not yet know why this phenomenon occurred in four patients but not in others with the same disease. We speculate that the hyperplastic breast tissue produces high levels of PSA under stimulation by steroid hormones. It is also possible that, in addition to the increased production, increased leakage of PSA may also occur in these tissues, a situation analogous to prostate cancer. Alternatively, aberrant expression of PSA, due to mutations in the promoter region of this gene may be the cause of high PSA. Clearly, more work is necessary to understand this phenomenon better.

We have already shown that PSA is produced by normal as well as hyperplastic and cancerous breast tissue (Yu et al, 1996). We demonstrated that total PSA levels show a significant decreasing trend with age, especially in hirsute women, women with benign breast diseases and women with breast cancer. This finding is in accord with data of tissue levels of PSA in breast cancer and PSA levels in nipple aspirate fluid. We found higher PSA levels in tumours and nipple aspirate fluids from younger women (Yu et al, 1994*a*; Sauter et al, 1996). These data suggest that PSA is regulated by ovarian steroids premenopausaly and probably by adrenal steroids post-menopausaly.

We found higher levels of total PSA in serum of women with DCIS and cancer patients with more children. The same trends were observed for PSA subfractions.

With the method used, only sera with total PSA > 15 ng $l^{-1}$ could be assessed for PSA subfractions. This limited our sample pool suitable for fractionation by HPLC from 632 to 50 sera. Remarkable among our findings was the observation that none of the sera from patients without breast pathology (*n* = 15; ten from blood donors; five from hirsute patients) had free PSA as the major molecular form. However, 44% of patients with cancer (presurgical sera) and 58% of patients with benign breast diseases had serum free PSA as the major molecular form. These findings confirm our preliminary observations (Melegos and Diamandis, 1996). These data allow us to speculate that free PSA appears to be abnormally elevated in diseases of the breast, including fibroadenomas and cancer. We propose that free PSA or the ratio of free/bound PSA, in contrast to total PSA, may have potential for the diagnosis of benign and malignant breast diseases. This issue should be examined in more detail, and with more patients, when new methods for free PSA analysis, without the need for HPLC, emerge as practical tools (Lilja et al, 1991; Cuny et al, 1996). Clearly, we would need PSA assays that can measure reliably at least 0.5 ng $l^{-1}$ of free or total PSA. Such assays are now being developed in our laboratory.

The underlying mechanism of free PSA increase in breast pathologies is unknown. We previously speculated that free PSA may be a mutant molecule but recent data do not support this hypothesis (Tsuyuki et al, 1997). Free PSA may be an inactive proenzyme, secreted by pathological tissue or a nicked form that is inactivated by an endopeptidase (McCormack et al, 1994; Zhang et al, 1995).

In conclusion, we have presented evidence for increased F/B PSA ratios in serum of patients with benign and malignant breast diseases. Refinements of these new findings may lead to the development of simple biochemical tests for diagnosis and monitoring of breast diseases including breast cancer.

### ABBREVIATIONS

PSA, prostate-specific antigen; ACT, $\alpha_1$-antichymotrypsin; A2M, $\alpha_2$-macroglobulin; HPLC, high-performance liquid chromatography; ER, oestrogen receptor; PR, progesterone receptor; F/B PSA, free/bound PSA; BPH benign prostatic hyperplasia;

© Cancer Research Campaign 1997 

PC, prostate cancer, DCIS, ductal carcinoma in situ; BBD, benign breast diseases.

### REFERENCES

Ouny C, Luong P, Kramp W, Sharp T and Soriano TF (1996) Evaluation of a two site immunoradiometric assay for measuring noncomplexed (free) prostate-specific antigen. *Clin Chem* **42**: 1243–1249

Diamandis EP and Yu H (1995) New biological functions of prostate-specific antigen? *J Clin Endocrinol Metab* **80**: 1515–1517

Diamandis EP, Yu H and Lopez-Otin C (1996) Prostate specific antigen – a new constituent of breast cyst fluid. *Breast Cancer Res Treat* **38**: 259–264

Ferguson RA, Yu H, Kalyvas M, Zammit S and Diamandis EP (1996) Ultrasensitive detection of prostate-specific antigen by a time-resolved immunofluorometric assay and the ImmuliteR immunochemiluminiscencent third-generation assay: potential applications in prostate and breast cancers. *Clin Chem* **55**: 675–684

Giai M, Rogana R, Ponzone R, Katsaros D, Levesque MA and Diamandis EP (1995) Prostate-specific antigen in serum of women with breast cancer. *Br J Cancer* **72**: 728–731

Leionen J, Loevgren T, Vornanen T and Stenman U (1993) Double-labeled time-resolved immunofluorometric assay of prostate-specific antigen and of its complex with $\alpha_1$-antichymotrypsin. *Clin Chem* **39**: 2098–2103

Levesque M, Yu H and Diamandis EP (1995) Prostate-specific antigen expression by various tumors. *J Clin Lab Anal* **9**: 123–128

Lilja H, Christensson A, Dahlém U, Matikainen M, Nilsson O, Petterson K and Loevgren T (1991) Prostate-specific antigen in serum occurs predominantly in complex with $\alpha_1$-antichymotrypsin. *Clin Chem* **37**: 1618–1625

Lilja H, Bjork T, Abrahamsson P-A, Stenman UH, Shaw N, Dowell B, Oesterling JE, Petterson K, Piironen T and Loevgren T (1994) Improved separation between normals, benign prostatic hyperplasia (BPH), and carcinoma of the prostate (CAP) by measuring free (F), complexed (C) and total concentrations (T) of prostate specific antigen. *J Urol* **151** (suppl.): 400A

Luderer A, Chen Y, Soriano T, Kramp W, Carlson G and Cuny C (1995) Measurement of the proportion of free to total prostate specific antigen (PSA) improves diagnostic performance of PSA in the diagnostic gray zone of total PSA 4 to 10 ng/mL. *Urology* **46**: 187–194

McCormack RT, Rittenhouse HG, Finlay JA, Sokoloff RL, Wang TJ, Wolfert RL and Oesterling JE (1994) Molecular forms of prostate-specific antigen and the human kallekrein gene family: a new era. *Urology* **45**: 729–744

Melegos DN and Diamandis EP (1996) Diagnostic value of molecular forms of prostate specific antigen for female breast cancer. *Clin Biochem* **29**: 193–200

Melegos DN, Yu H, Ashok M, Wang C, Stanczyk F and Diamandis EP (1997) Prostate specific antigen in female serum – A potential new marker of androgen excess. *J Clin Endocrinol Metab* **82**: 777–780

Sauter ER, Daly M, Lenahan K, Ehya H, Engstrom PF, Sorling A, Bonney G, Yu H, Diamandis EP (1996) Prostate specific antigen in nipple aspirate fluid correlate with breast cancer risk. *Cancer Epidemiol Biomarkers Prevent* **5**: 967–970

Stenman UH, Leionen J, Alfthan H, Rannikko S, Tuhkanen K and Alfthan O (1991) A complex between prostate-specific antigen and $\alpha_1$-antichymotrypsin is the major form of prostate specific antigen in serum of patients with prostate cancer: assay of the complex improves clinical sensitivity for cancer. *Cancer Res* **51**: 222–226

Tsuyuki D, Grass L and Diamandis EP (1997) Frequent detection of mutations in the 5′ flanking region of the prostate specific antigen gene in female breast cancer. *Eur J Cancer* (in press)

Vessella R, Noteboom J and Lange P (1992) Evaluation of Abbott IMx automated immunoassay of prostate-specific antigen. *Clin Chem* **38**: 2044–2054

Yu H and Diamandis EP (1995*a*) Prostate-specific antigen in milk of lactating women. *Clin Chem* **41**: 54–58

Yu H and Diamandis EP (1995*b*) Prostate-specific antigen immunoreactivity in amniotic fluid. *Clin Chem* **41**: 204–210

Yu H, Diamandis EP and Sutherland DJA (1994*a*) Immunoreactive prostate-specific antigen levels in female and male breast tumors and its association with steroid hormone receptors and patient age. *Clin Biochem* **27**: 75–79

Yu H, Diamandis EP, Zarghami N and Grass L (1994*b*) Induction of prostate specific antigen production by steroids and tamoxifen in breast cancer cell lines. *Breast Cancer Res Treat* **32**: 291–300

Yu H, Diamandis EP, Levesque M, Giai M, Roagna R, Ponzone R, Sismondi P, Monne M and Croce C (1996) Prostate specific antigen in breast cancer, benign breast disease and normal breast tissue. *Breast Cancer Res Treat* **40**: 171–178

Yu H, Giai M, Diamandis EP, Katsaros D, Sutherland DJA, Levesque MA, Roagana R and Sismondi P (1995*a*) Prostate-specific antigen is a new favorable prognostic indicator for women with breast cancer. *Cancer Res* **55**: 2104–2110

Yu H, Diamandis EP, Monne M and Croce CM (1995*b*) Oral contraceptive-induced expression of prostate specific antigen in female breast. *J Biol Chem* **270**: 6615–6618

Zarghami N, Grass L, Diamandis EP (1997) Steroid hormone regulation of prostate specific antigen gene expression in breast cancer. *Br J Cancer* **75**: 579–588

Zhang W-M, Leionen J, Kalkkinen N, Dowell B and Stenman U-H (1995) Purification and characterization of different molecular forms of prostate-specific antigen in human seminal fluid. *Clin Chem* **41**: 1567–1573

© Cancer Research Campaign 1997